# The Effect of Elective Ligation of the Arteriovenous Fistula on Cardiac and Renal Functions in Kidney Transplant Recipients

**DOI:** 10.34067/KID.0000000000000198

**Published:** 2023-06-26

**Authors:** Grégoire Masson, Tommaso Viva, Justine Huart, Laurent Weekers, Catherine Bonvoisin, Antoine Bouquegneau, Sylvie Maweja, Etienne Hamoir, Laurence Seidel, Hans Pottel, Patrizio Lancellotti, François Jouret

**Affiliations:** 1 Division of Nephrology, Department of Internal Medicine, University of Liège Hospital (ULiège CHU), Liège, Belgium; 2 Division of Cardiology, Department of Internal Medicine, University of Liège Hospital (ULiège CHU), Liège, Belgium; 3 Unit of Cardiovascular Sciences, Groupe Interdisciplinaire de Génoprotéomique Appliquée (GIGA), Cardiovascular Sciences, University of Liège (ULiège), Liège, Belgium; 4 Division of Abdominal Surgery and Transplantation, Department of Surgery, University of Liège Hospital (ULiège CHU), Liège, Belgium; 5 Department of Biostatistics, University of Liège Hospital (ULiège CHU), Liège, Belgium; 6 KU Leuven Kulak, Department of Public Health and Primary Care, University of Leuven, Kortrijk, Belgium

**Keywords:** arteriovenous fistula, kidney transplantation, echocardiography, left ventricular hypertrophy, BP, eGFR, cardiovascular, hypertension, kidney dysfunction, transplantation, vascular access

## Abstract

**Key Points:**

- Surgical AVF ligation in KTRs is associated with a significant increase in diastolic BP while systolic BP remains stable.
- AVF closure in KTRs leads to an improvement of LV and LA morphology and a decrease in serum NT-proBNP levels.
- There is no significant effect of AVF ligation on kidney allograft function: The eGFR remains stable over time.

**Background:**

Kidney transplantation is considered as the best kidney replacement therapy, and arteriovenous fistula (AVF) is the preferred vascular access for hemodialysis. The systematic ligation of a functioning AVF in stable kidney transplant recipients (KTRs) remains debatable.

**Methods:**

In this prospective study, we investigated the hemodynamic effect of the surgical closure of AVF in KTRs. Forty-three KTRs underwent an ambulatory BP monitoring before surgical closure of AVF (T0) and 12 months later (M12), as well as measurement of serum cardiac biomarkers (*i.e.*, soluble suppression of tumorigenicity 2, N-terminal pro b-type natriuretic peptide [NT-proBNP], and galectin-3). Serum tests were also performed 6 months after AVF closure (M6). An echocardiographic examination was performed at each time point. All serum creatinine values were collected to compare the individual eGFR slopes before versus after AVF closure. The latest measure of the AVF flow before kidney transplantation was recorded.

**Results:**

Diastolic BP significantly rose from T0 to M12: +4.4±7.3 mm Hg (*P* = 0.0003) for 24h, +3.8±7.4 mm Hg (*P* = 0.0018) during the day, and +6.3±9.9 mm Hg (*P* = 0.0002) during the night, leading to an increased proportion of KTRs with European Society of Hypertension (ESH)-defined arterial hypertension after AVF ligation. No change was observed for systolic BP. NT-proBNP significantly dropped between T0 and M6 (345 [190; 553] to 230 [118; 458] pg/ml, *P* = 0.0001) and then remained stable from M6 to M12 while suppression of tumorigenicity 2 and galectin-3 levels did not change from T0 to M12. We observed a significant decrease in left ventricular (LV) end-diastolic volume, LV end-systolic volume, LV mass, interventricular septum diameter, left atrial volume, and tricuspid annular plane systolic excursion from T0 to M6 and then a stability from M6 to M12. LV ejection fraction and eGFR slope remained stable during the whole study. These observations remained unchanged after adjustment for AVF flow.

**Conclusion:**

The closure of a patent AVF in KTRs is associated with elevation of diastolic BP, drop in serum NT-proBNP levels, reduction of left ventricular and atrial dimensions, and stability of eGFR slope.

## Graphical abstract

**Figure:**
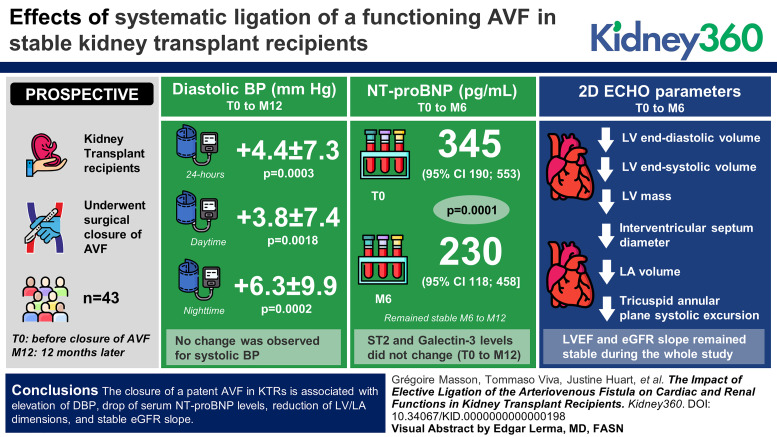

## Introduction

Since its first description by Brescia and Cimino in 1966,^1^ arteriovenous fistula (AVF) has been rapidly regarded as the best vascular access for patients with ESKD undergoing hemodialysis, as stated by the Kidney Dialysis Outcome Quality Initiative of the National Kidney Foundation,^2^ by the Kidney Disease: Improving Global Outcomes,^3^ and by the European Society for Vascular Surgery.^4^ AVF carries a lower risk of infection and thrombosis in comparison with catheters. Although the indications and surgical techniques of AVF creation are rather consensual, limited literature exists regarding the management of a functioning AVF in kidney transplant recipients (KTRs) with a stable and sufficient renal function. Kidney transplantation (KTx) represents the best option for patients with ESKD, with significant improvement of survival and quality of life compared with dialysis. However, the global survival of a kidney graft at 5 years remains approximately 70%.^5^ The main reasons for graft loss are acute rejection and chronic graft dysfunction. The pathophysiology of the latter is poorly understood and includes infectious, immunological, and hemodynamic causes. Among the hemodynamic causes, the effect of AVF ligation on renal graft function remains controversial. In most patients, AVF remains functional after a successful KTx. The persistence of a functional AVF at 1 year after KTx has been retrospectively associated with a lower eGFR and an increased risk of graft loss.^6^ Whereas in another publication, a significant acceleration of eGFR decline over the 12 months after the closure of a functioning AVF in KTRs^7^ has been reported in a retrospective monocentric cohort. By contrast, AVF closure has been associated with cardioprotective effects in prospective studies with a rather limited number of KTRs.^8–11^ These observations were not confirmed in other trials in which AVF persistence for prolonged periods after KTx had minor consequences on cardiac morphology and function.^12–14^ Thus, on the basis of this highly controversial literature, the systematic ligation of a functioning AVF remains debatable in stable KTRs.

The protocol in our center is to surgically ligate AVFs 1 year after KTx with the patient's approval if the eGFR is stable and higher than 45 ml/min per 1.73 m^2^ or if there are symptoms or signs of overt heart failure (HF). We conducted a prospective single-center trial to monitor the variations in renal graft function, cardiac hemodynamics, and BP up to 4 years after AVF surgical ligation after KTx.

## Patients and Methods

### Patients

From January 2017 to January 2022, 43 patients were recruited and followed up after a signed informed consent at the University of Liège Hospital (ULiège CHU) in Liège, Belgium. This observational study was approved by the Institutional Review Board of ULiège CHU (Reference number: B707201630146), in adherence to the Declaration of Helsinki. The clinical and research activities being reported are consistent with the principles of the Declaration of Istanbul as outlined in the Declaration of Istanbul on Organ Trafficking and Transplant Tourism. Between 2017 and 2019, we recruited up to 15 KTRs per year, but the coronavirus disease 2019 pandemic interrupted this enrollment from 2020 to 2021. In subset populations, the follow-up was extended to 24 months (M24) after AVF ligation in 15 patients, to 36 months (M36) in eight patients, and to 48 months (M48) in ten patients.

### Design of the Prospective Study

Before the surgical ligation of AVF (T0) and 12 months after the surgery (M12), all patients had a 24-hour ambulatory BP monitoring. The ambulatory BP monitoring was performed using a Mobil-O-Graph 24h Pulse Wave Analysis Monitor (IEM GmbH, Aachen, Germany). BP was measured every 20 minutes during the day and every 30 minutes during the night. Mean daytime and nighttime systolic (SBP) and diastolic (DBP) BP levels were calculated on the basis of self-declared awake and asleep periods. A patient was categorized as dipper when the night–day SBP ratio was ≤0.9 or nondipper when the night–day SBP ratio was >0.9. Serum cardiac biomarkers (*i.e.*, soluble suppression of tumorigenicity 2 [ST2], N-terminal pro b-type natriuretic peptide [NT-proBNP], and galectin-3) were measured at T0, at 6 months after AVF closure (M6), and at M12. ST2 was measured by ELISA, NT-proBNP by the chemiluminescent microparticle method on Alinity I (Abbott, Abbott Park, IL), and galectin-3 levels by Alinity C (Abbott, Abbott Park, IL). We prospectively collected all serum creatinine (SCr) values regularly measured during the conventional follow-up of KTRs. SCr was measured by the enzymatic method (Alinity C, Abbott, Abbott Park, IL). The eGFR was determined using the Modification of Diet in Renal Disease (MDRD) equation.^15^ The MDRD equation is currently regarded as the most accurate estimation of GFR in KTRs.^16^ Transthoracic echocardiograms were performed by using Vivid S70 (GE Healthcare, Chicago, IL) at T0, M6, and M12. We measured left ventricular (LV) end-diastolic diameter (LVEDD), LV end-systolic diameter, LV end-diastolic volume (LVEDV), LV end-systolic volume, LV mass, interventricular septum diameter, posterior wall diameter, relative wall thickness, stroke volume (SV), cardiac output (CO), cardiac index (CI), LV ejection fraction (LVEF), left atrial volume (LAV), E/A ratio, and tricuspid annular plane systolic excursion (TAPSE). LVEDD, LV end-systolic diameter, LVEDV, LV end-systolic volume, LV mass, SV, and LAV were indexed to body surface area. Data on AVF flow (QAVF) before access ligation were not available as the baseline (T0) parameter, but the latest QAVF that was measured before KTx was recorded.

### Statistical Analyses

Descriptive statistics were expressed as mean and SD or as median and quartiles (first quartile–third quartile) for continuous variables and as frequency tables for qualitative variables. The paired Student *t*-test and Wilcoxon signed-rank test were used to compare the means of continuous variables and medians between T0 and M12, respectively. The McNemar test was used to compare the proportions of qualitative variables between T0 and M12. The evolution of the parameters along time was analyzed by the general linear mixed model. In the case of dissymmetric distribution, a logarithm transformation was applied. For binary distribution, we used a general estimating equation regression model, also considering repeated measures for KTRs. Concerning the renal function, we excluded SCr values during the first 3 months after KTx to avoid the usual fluctuations of renal function in the immediate post-KTx period. After this period, all available SCr values were included in our analyses. Linear splines for MDRD versus time were used with one knot at the time of AVF closure, allowing separate regressions of MDRD for each period (before and after AVF closure). Time was balanced before and after AVF closure with at least ten observations per patient. Slopes and intercepts before and after AVF closure were compared using paired *t*-tests. Results were considered significant at the 5% significance level (*P* < 0.05). All analyses were performed with SAS 9.4 (SAS Institute, Cary, NC).

## Results

Our cohort study involved 43 KTRs (Table 1) with a female/male ratio of 15/28. The mean age was 51.3±15.4 years. The mean cold ischemia time reached 754 (533; 901) minutes, with a global rate of delayed graft function requiring dialysis of 2.9%. Expanded criteria donors concerned 16.7% of all KTxs. AVFs were in most patients located on the left (72.1%) forearm (88.4%). 83.3% of our KTRs had an eGFR of >45 ml/min per 1.73 m^2^ before AVF surgical ligation. The other indications were cosmetic, and only one ligation was performed for a cardiologic cause. AVF closure took place after a median time of 560 (447; 789) days after KTx. QAVF was measured before KTx in 30 patients (69.8%): 750 (750; 1320) ml/min. More than 80% of KTRs had antihypertensive treatment at baseline, with a median of two drugs per patient. Five patients had more than four drugs.

**Table 1.** Characteristics of the population

Parameters	All Patients (*n*=43)
**Recipients**	
Age (yr)	51.3±15.4
Sex (F/M)	15/28
BMI at KTx (kg/m^2^)	25.9±4.5
Duration of dialysis (d)	562 (298; 841)
QAVF before KTx (ml/min)	750 (750; 1320)
AVF ligation after KTx (d)	560 (447; 789)
**Donor**	
Age (yr)	44.4±11.7
Sex (F/M)	18/25
BMI (kg/m^2^)	25.7±4.4
DBD/DCD/LD (%)	74/19/7
ECD (%)	16.7
**Transplant**	
CIT (min)	754 (532.5; 901)
dDGF (%)	2.9

Plus–minus values are mean±SD. F, female; M, male; BMI, body mass index; KTx, kidney transplantation; QAVF, AVF flow; AVF, arteriovenous fistula; DBD, donor after brain death; DCD, donor after circulatory death; LD, living donor; ECD, expanded criteria donor; CIT, cold ischemic time; dDGF, dialysis-based definition delayed graft function.

### Evolution of BP Levels after AVF Ligation in KTRs

DBP significantly rose from T0 to M12: +4.4±7.3 mm Hg (*P* = 0.0003) for 24h-DBP, +3.8±7.4 mm Hg (*P* = 0.0018) for daytime DBP, and +6.3±9.9 mm Hg (*P* = 0.0002) for nighttime DBP (Table 2). SBP and heart rate remained stable over time. These results were similar with or without adjustment for QAVF (Table 2). The percentage of KTRs who fulfilled the hypertension criteria of the European Society of Hypertension (*i.e.*, mean 24h-DBP ≥80 mm Hg) increased from 39.5% to 65.1% (*P* = 0.0045). The proportion of patients with a physiological dipping, decreased from 12.6% at T0 to 8.7% at M12 (*P* = 0.041). These observations persisted until M24, M36, and M48 in the subgroups with extended follow-up. More particularly, the 24h-DBP levels increased from 78.8±8.7 mm Hg (at T0) to 78.1±7.9 mm Hg (at M24), 86.1±13.9 mm Hg (at M36), and 83.3±6.2 mm Hg (at M48) (*P* = 0.007). The percentage of KTRs treated with antihypertensive treatment remains stable during the whole study (81.4% at T0, 76.74% at M12, 86.67% at M24, 75% at M36, and 90% at M48). The median number of antihypertensive drugs was also constant around two (*P* = 0.93).

**Table 2.** 24h Ambulatory BP monitoring and cardiac biomarkers

Parameters	T0		M12		*P* Value	*P* Value *QAVF
24h SBP (mm Hg)	131±12.6		129.5±12.4		0.61	0.52
24h DBP (mm Hg)	78.8±8.7		83.2±9.0		0.0003	0.004
Daytime SBP (mm Hg)	133±12.3		132±12.1		0.47	0.53
Daytime DBP (mm Hg)	81.4±8.9		85.2±9.2		0.0018	0.005
Nighttime SBP (mm Hg)	123±14.2		124±15.9		0.55	0.98
Nighttime DBP (mm Hg)	71.2±9.8		77.5±10.7		0.0002	0.007
24h heart rate (bpm)	72±11.1		72±10.5		0.85	0.57
	T0		M6	M12			
NT-proBNP (pg/ml)	345 (190; 553)	230 (118; 458)		191 (129; 344)	0.0001	0.022
ST2 (ng/ml)	23.8 (19.9; 29.8)	22.8 (19.8; 27.8)		23.5 (19.2; 32.7)	0.92	0.63
Galectin-3 (ng/ml)	19.5 (13.9; 23.6)	17.4 (13.4; 21.2)		18.7 (13.6; 24.1)	0.30	0.21

24h ambulatory BP monitoring values are mean±SD. Cardiac biomarkers are medians (first quartile; third quartile). T0, before the surgical ligation of the AVF; M12: 12 months after the surgery; *QAVF, *P* value after adjustment for QAVF (*n*=30 cases); QAVF, AVF flow; SBP, systolic BP; DBP, diastolic BP; M6: 6 months after the surgery; NT-proBNP, N-terminal pro b-type natriuretic peptide; ST2, suppression of tumorigenicity 2 marker.

### Evolution of Serum Levels of Cardiac Biomarkers after AVF Ligation in KTRs

**Figure 1.**
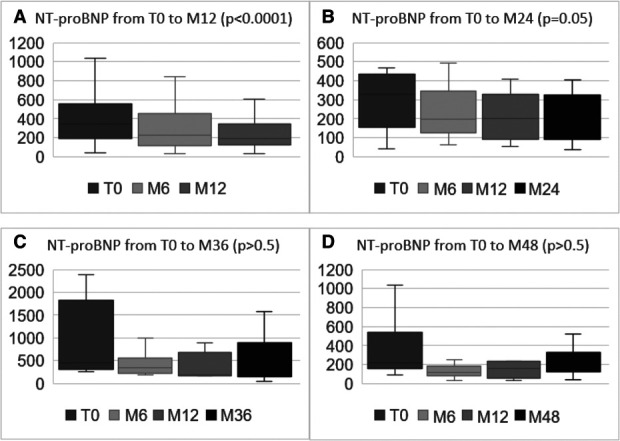
**Evolution of NT-proBNP levels.** Changes in NT-proBNP serum levels in the whole cohort (*n*=43) from T0 to M12 after KTx (A); in patients (*n*=15) who had follow-up to 24 months after KTx (B); in patients (*n*=8) who had follow-up to 36 months after KTx (C); and in patients (*n*=10) who had follow-up to 48 months after KTx (D). Changes in means and 95% confidence intervals showed a significant decrease of NT-proBNP from T0 to M12 (*P* < 0.0001) with the general linear mixed model. NT-proBNP serum levels are expressed in pg/ml. KTx, kidney transplantation; M6 and M12: 6 and 12 months, respectively, after the surgery; NT-proBNP, N-terminal pro b-type natriuretic peptide; T0, before the surgical ligation of the AVF.

NT-proBNP significantly dropped between T0 (345 [190; 553] pg/ml) to M6 (230 [118; 458] pg/ml; *P* < 0.05 with or without adjustment for QAVF) and then remained stable from M6 to M12 (Table 2). No significant change was observed beyond M12: 251 (91; 326) pg/ml at M24, 272 (165; 740) pg/ml at M36, and 203 (131; 276) pg/ml at M48 (Figure 1). ST2 and galectin-3 remained globally stable during the whole study, with a significant trend toward decreased levels of ST2 in the long term (17.7 ng/ml at M48, *P* = 0.0023).

### Evolution of Renal Function after AVF Ligation in KTRs

**Figure 2.**
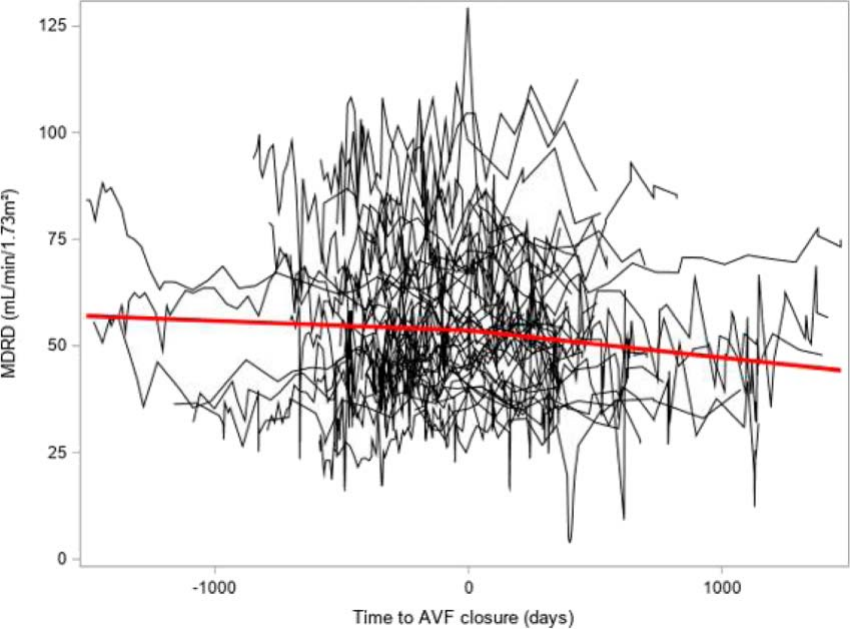
**Evolution of eGFR according to MDRD.** MDRD eGFR slopes (in ml/min per 1.73 m^2^) of kidney transplant recipients before versus after closure of the AVF. The slopes do not differ from each other through time (*P* = 0.248). AVF, arteriovenous fistula; MDRD, Modification of Diet in Renal Disease.

Two patients were excluded from the analysis because of a lack of a sufficient number of SCr values. Among 41 patients, there were 63.4% with a negative MDRD-eGFR slope before AVF closure and 46.3% with a negative MDRD-eGFR slope after AVF closure. The MDRD-eGFR slope was −0.0278 ml/min per 1.73 m^2^ per year (−0.339; 0.326) before AVF closure and −1.053 ml/min per 1.73 m^2^ per year (−4.5656; 2.313) after AVF closure. The median time was 439 (360; 539) days before AVF closure and 370 (336; 497) days after AVF closure. There was no significant difference between slopes before and after AVF closure in the whole cohort (*P* = 0.25). The mean of the paired differences between slopes in one given patient was −0.001 ml/min per 1.73 m^2^ per year with 95% confidence interval −0.094 to +0.072. Figure 2 depicts the eGFR slopes according to the MDRD equation.

### Evolution of Echocardiographic Parameters after AVF Ligation in KTRs

After AVF closure, we observed a significant decrease in LVEDD, LVEDV, interventricular septum diameter, SV, LAV, CO, CI, and E/A ratio from T0 to M6. LV mass and LV mass index decreased to 32 g (45; 34) and 16.5 g/m^2^ (18.2; 13.6) from T0 to M12 (*P* < 0.0001), respectively. There was no significant change in LVEF. Right heart parameters, such as basal right ventricle end-diastolic diameter, pulmonary arterial systolic pressure, and TAPSE, dropped from T0 to M12. All of these data are summarized in Table 3. The long-term follow-up showed the persistence of the echocardiographic changes described above. The general linear mixed model demonstrated that the LAV index correlated with the concentration of serum NT-proBNP (*P* = 0.0004). The LAV index and LVEDD increased as a function of ST2 (*P* = 0.036 and *P* = 0.0003, respectively) and galectin-3 (*P* < 0.0001 and *P* = 0.017, respectively).

**Table 3.** Summary of changes in echocardiograms

Parameters	T0	M6	M12	*P* Value
LVEDD (mm)	50±5.07	47.4±4.92	47±5.94	<0.0001
LVEDD index (mm/m^2^)	26.6±3.20	25.3±3.11	25.1±3.39	<0.0001
LVESD (mm)	33.4±5.54	31.2±4.80	33.0±5.63	0.087
LVESD index (mm/m^2^)	17.8±3.32	16.6±3.10	17.8±3.29	0.10
LVEDV (ml)	125±30.9	106±24.9	105±21.7	<0.0001
LVEDV index (ml/m^2^)	65.8±14.5	56.1±11.1	55.2±10.1	<0.0001
LVESV (ml)	49±16.9	42.0±13.0	44.0±13.2	0.012
LVESV index (ml/m^2^)	25.7±8.36	22.1±6.28	23.0±6.14	0.012
LV mass (g)	187±55.6	154±42.3	155±50.7	<0.0001
LV mass index (g/m^2^)	97.9±24.7	81.1±17.3	81.4±22.1	<0.0001
Interventricular septum diameter (mm)	11.6±2.06	10.8±2.06	10.9±1.78	0.0003
PW diameter (mm)	8.45±1.95	7.80±1.67	7.90±1.55	0.12
RWT	0.34±0.08	0.33±0.081	0.34±0.063	0.86
SV (ml)	97.1±18.9	89.4±29.9	86.5±22.1	0.0061
SV index (ml/m^2^)	51.9±9.39	46.7±12.4	45.5±10.4	0.0034
CO (L/min)	6.49±1.15	5.61±2.12	5.98±1.95	0.0054
CI (L/min per m^2^)	3.48±0.66	3.00±0.94	3.15±0.89	0.0079
Ejection fraction (%)	61.5±6.51	60.5±8.11	58.2±8.74	0.078
Left atrial volume (ml)	72±28.1	55.5±16.1	60.2±22.4	<0.0001
Left atrial volume index (ml/m^2^)	37.5±12.6	29.3±6.65	31.3±9.97	<0.0001
E/A ratio	1.13±0.34	0.94±0.30	0.94±0.34	<0.0001
TAPSE (mm)	23.4±4.77	21.4±3.84	21.2±4.24	0.011
Basal RV end-diastolic diameter (mm)	38.5±4.55	36.7±3.90	36±4.12	0.015
Pulmonary arterial systolic pressure (mm Hg)	26.9±7.17	24.4±6.11	21.9±6.42	0.0037

Plus–minus values are mean±SD. T0, before the surgical ligation of the AVF; M6 and M12: 6 and 12 months, respectively, after the surgery; LVEDD, left ventricular end-diastolic diameter; LVESD, left ventricular end-systolic diameter; LVEDV, left ventricular end-diastolic volume; LVESV, left ventricular end-systolic volume; LV, left ventricle; PW, posterior wall; RWT, relative wall thickness; SV, stroke volume; CO, cardiac output; CI, cardiac index; TAPSE, tricuspid annular plane systolic excursion; RV, right ventricle.

## Discussion

In this prospective and systematic study, the surgical ligation of a functioning AVF causes the isolated elevation of DBP during both daytime and nighttime, with no significant influence on SBP. From a clinical point of view, such an increased DBP leads to the diagnosis of hypertension in a significantly higher proportion of initially normotensive patients according to ESH criteria.^17^ Still, serum levels of NT-proBNP drop concomitantly with long-term improvements of the US-based cardiac parameters. No significant effect on kidney function was noted in our prospective cohort.

A survey realized in a Polish KTx center mentioned that only one-fourth of KTRs have ever considered AVF closure, mainly for cosmetic reasons.^18^ In an American retrospective study, more than 16.000 medical files were reviewed: Only 4.6% of KTRs had AVF surgical closure, with a high variability between centers, and the systemic benefits of AVF ligation, including graft failure and all-cause mortality, were minimal.^19^ In our center, we propose a surgical AVF ligation to KTRs 1 year after KTx if eGFR is >45 ml/min per 1.73 m^2^, if the risk of recurrence of initial nephropathy is low or zero, if there is no history of rejection or graft loss, if the donor-specific antibodies are absent, and finally if the adherence to medication is optimal. Owing to a lack of standardization or guidelines, eGFR >45 ml/min per 1.73 m^2^ was chosen arbitrarily. Importantly, the motivation of the patient regarding AVF ligation also influences the final surgical decision. Magnetti *et al.* showed that AVF closure in 22 KTRs significantly improved the kidney graft resistivity index (from 0.71 [0.66–0.74] before AVF closure to 0.66 [0.61–0.69] 6 months later, *P* < 0.001), possibly by a better graft perfusion.^20^ Indeed, one may speculate that the AVF-associated low resistance attenuates the arterial stiffness and wall pressure,^21^ thereby improving organ perfusion. Central arteriovenous anastomosis has been proposed to treat uncontrolled hypertension following this physiological hypothesis of reduced vascular resistances.^22^

Management of isolated diastolic hypertension (IDH) is debated. First, IDH was regarded as part of the physiological aging phenomenon because of arterial stiffness. However, a Finnish prospective study and two Asian meta-analyses finally demonstrated that IDH was associated with a higher cardiovascular mortality in the general population.^23–25^ As stated by ESH/European Society of Cardiology 2018 guidelines,^17^ lifestyle changes and antihypertensive drugs should lower office DBP to <90 mm Hg (recommendation IA), and maybe toward 80 mm Hg (recommendation IIaB). No specific management is proposed for KTRs. The isolated rise of DBP after AVF ligation could be related to a healthier vascular system because of the rather young age of KTRs. Unfortunately, no data were available concerning arterial stiffness or renal resistive index in our cohort.

AVF ligation induces cardiac morphological changes by suppressing the hemodynamically significant high-flow state. A prophylactic surgical high-flow AVF ligation (>1500 ml/min) has been previously shown to prevent high-output HF (0% versus 38.3% in the control group, *P* = 0.013).^26,27^ In our present series, 7 of 30 patients (23.3%) had a QAVF higher than 1500 ml/min. In a single-center randomized controlled trial (AVF ligation versus no ligation), the reduction of the LV mass index, as well as LV and LA volumes, CO, CI, but not LVEF, after 6 months from AVF closure was reported using cardiac magnetic resonance imaging.^28^ Notably, no change in cardiac parameters was observed in control patients with no AVF ligation, which emphasizes the statistical robustness of comparing each patient to him/herself at T0. Salehi *et al.* showed that the regression of cardiac magnetic resonance imaging–measured LV mass and LV mass index persists 5 years after AVF ligation.^11^ However, the cardiac parameters did not fully return to normal values because a residual concentric remodeling of LV geometry remained.^8^ Our results are consistent with these findings and confirm the LV and LA reverse remodeling after 6 months of AVF closure. We also demonstrate the stability of this condition after 12 months. Regression of LV hypertrophy in KTRs has been associated with better survival rates and less cardiovascular events in a prospective *post hoc* analysis of two randomized control trials where changes in LV mass were observed after either starting angiotensin-converting enzyme inhibitors versus no therapy or add-on therapy with everolimus over cyclosporine.^29^ LVEF was stable over the duration of the study. It is the most practical and frequently used parameter to assess LV systolic function despite the limitation of its pre- and after-load dependence. Note that the high flow generated by a functioning AVF increases the pre-load, which probably leads to an overestimation of LV systolic function compared with the “after AVF closure” status. Therefore, the systolic function factually increases, but this effect is masked by the high-flow state before AVF ligation. Moreover, the DBP elevation after AVF ligation probably increases the after-load, thereby reducing LVEF. The QAVF was routinely measured before KTx in 30 patients of our cohort. The changes in BP levels and biomarker serum concentrations after AVF ligation were not significantly affected after statistical adjustment for QAVF. Note that the AVF flow rate does not fully respond to Poiseuille flow theory. First, the flow is pulsatile. Second, the modified vascular anatomy induces complex flow patterns. Furthermore, Doppler ultrasound may overestimate the blood flow in tortuous segments of the AVF.^30,31^

A reduction in right ventricular longitudinal systolic function, assessed by TAPSE, after AVF closure was demonstrated. This is probably related to the fact that TAPSE is a preload-dependent parameter. Pulmonary hypertension is defined by a mean pulmonary arterial pressure of ≥25 mm Hg and is often seen in patients with ESKD, especially those with (high-flow) AVF. Volume overload, increased vascular tone, and underlying HF can contribute to or exacerbate pulmonary hypertension. As seen in our study, pulmonary arterial systolic pressure seems to be improved after AVF ligation, which may lead to improved cardiac outcomes.

The natriuretic peptides NT-proBNP and BNP are synthetized by atrial and ventricular myocardia, but the main stimulus is the stretch of ventricular cardiomyocytes. Pre–AVF ligation NT-proBNP is determined by the myocardial stretch (wall stress) induced by the chronic high-flow state. The normalization of the flow after AVF ligation causes the drop in NT-proBNP together with the reductions in LVEDV and LAV.^32,33^ The natriuretic peptides are used in routine clinical practice as initial screening tests to rule out HF in symptomatic patients. However, these biomarkers are not recommended to guide titration of therapy given the conflicting results of various trials.^34^ Our findings are in agreement with other reports that show that serum levels of NT-proBNP decrease after AVF ligation, in parallel with modifications of cardiac morphology and function.^35^ ST2 is a biomarker of fibrosis and inflammation and a novel biomarker to diagnose and risk-stratify HF and various cardiac diseases.^36^ A Spanish team demonstrated that a decrease in serum ST2 levels during the 2-week monitoring period was associated with less HF episodes, independently of serum NT-proBNP levels.^37^ Galectin-3 is a *β*‐galactoside–binding lectin leading to apoptosis, angiogenesis, inflammation, and, finally, myocardial fibrogenesis and HF. Galectin-3 is considered as a biomarker of the severity of heart fibrosis.^38^ Acute and chronic renal failure can increase serum galectin-3 levels.^39^ Furthermore, heart transplantation does not seem to change serum levels of galectin-3 of patients with HF.^40^ Both biomarkers are now approved by the Food and Drug Administration as complementary biomarkers to assess the global risk of HF. As mentioned above, AVF ligation was followed by cardiac functional and morphological changes, but no significant change was observed in serum galectin-3 and ST2 levels in KTRs. It is of interest to see a downward trend for serum levels of ST2 after 36 months in our cohort. These findings could be explained by persistent cardiac morphological abnormalities after AVF closure.^8^ To our knowledge, this is the first time serum ST2 and galectin-3 are measured in KTRs after AVF closure. Theoretically, the renal clearance may also influence serum ST2 and galectin-3 levels. We postulate that very small changes in cardiac morphology remaining after AVF closure can be sufficient to stimulate ST2 and galectin-3 production, but further investigations are needed.

The effect of AVF ligation on eGFR decline in KTRs remains highly debated. A meta-analysis showed that KTRs with ligated AVF had lower SCr values with a pooled mean difference of 0.10 (95% confidence interval, 0.04 to 0.17; *P* = 0.003) compared with patients with left-open AVF.^41^ A bias of indication may partly explain these findings because higher SCr values increase the probability of ESKD in KTRs with a soon-to-come need for vascular access and the subsequent decision to not ligate a functioning AVF in these patients. In a retrospective study individually comparing 114 KTRs with ligated AVF with themselves, the eGFR slopes were significantly different before (0.038 ml/min per month) versus after (−0.159 ml/min per month) AVF closure. One may extrapolate that the decline in kidney function after AVF ligation may be caused by lower pulmonary flow and decreased arterial oxygen content.^7^ Modifications in body composition, including edema-free weight and surface, may also interfere with eGFR assessment.^42^ In this prospective study of a limited number of patients, no significant difference was found when comparing the eGFR slopes before versus after AVF ligation. Large inter- and intra-individual variabilities of SCr values were observed. Comparatively measured GFR before and after surgery in large multicenter prospective studies may help resolve this debated question about the effect of AVF closure on kidney function decline.

In conclusion, AVF closure in KTRs is associated with improved LV and LA morphology and decreased serum NT-proBNP levels, but these positive cardiologic effects could be mitigated by an increase in DBP. No significant change in eGFR slopes and LVEF was observed after AVF ligation. Although our cohort is the largest one studied so far, the absence of a control group is the main limitation of our work. Note that Rao *et al.* demonstrated that the absence of AVF ligation in KTRs does not change any clinical, biological, or radiological parameters at 6 months.^28^ Scoring scales and standardizations are needed to better assess the indications for AVF closure, including the global cardiovascular risk. The surgical ligation of a patent AVF should only be considered in patients with stable and preserved renal function or in patients at risk of HF, and careful monitoring of DBP is recommended.

## Supplementary Material

SUPPLEMENTARY MATERIAL

## Data Availability

All data is included in the manuscript and/or supporting information.
