# Supplementary material for: The Effect of Elective Ligation of the Arteriovenous Fistula on Cardiac and Renal Functions in Kidney Transplant Recipients
Source: Kidney360. 2023 Jun 26;4(8):1130–8. doi: 10.34067/KID.0000000000000198 (PMC10476678; doi:10.34067/KID.0000000000000198)
Supplement: SUPPLEMENTARY MATERIAL [file kidney360-4-1130-s001.pdf]

SUPPLEMENTARY MATERIAL

Table of contents

- Supplementary Table S1. Baseline patient demographics and clinical characteristics for the CONFIRM, OT-0401, and REVERSE studies; ITT populations

Supplementary Table S1. Baseline patient demographics and clinical characteristics for the CONFIRM[13], OT-0401[11], and REVERSE[12] studies; ITT populations

	**CONFIRM**		**OT-0401**		**REVERSE**	
**Baseline characteristics**	**Terlipressin (n=199)**	**Placebo (n=101)**	**Terlipressin (n=56)**	**Placebo (n=56)**	**Terlipressin (n=97)**	**Placebo (n=99)**
Age (years), mean (SD)	54.0 (11.34)	53.6 (11.83)	50.6 (10.50)	52.9 (11.40)	55.8 (8.38)	54.8 (8.50)
Sex, male	120 (60.3)	59 (58.4)	41 (73.2)	39 (69.6)	52 (53.6)	67 (67.7)
Race						
American Indian or Alaskan Native	2 (1.0)	0 (0.0)	0 (0.0)	3 (5.4)	1 (1.0)	1 (1.0)
Asian	5 (2.5)	1 (1.0)	0 (0.0)	0 (0.0)	3 (3.1)	0 (0.0)
Black or African American	12 (6.0)	5 (5.0)	5 (8.9)	3 (5.4)	7 (7.2)	6 (6.1)
Native Hawaiian or Other Pacific Islander	0 (0.0)	0 (0.0)	0 (0.0)	1 (1.8)	0 (0.0)	0 (0.0)
White	177 (88.9)	94 (93.1)	51 (91.1)	49 (87.5)	85 (87.6)	92 (92.9)
Alcoholic hepatitis, present	81 (40.7)	39 (38.6)	20 (35.7)	20 (35.7)	20 (20.6)	25 (25.3)
Baseline sCr	199	101	56	56	97	99
Mean (SD), mg/dL	3.5 (1.01)	3.5 (1.06)	4.0 (2.19)	3.8 (1.17)	3.6 (1.05)	3.7 (1.11)
<3 mg/dL	79 (39.7)	40 (39.6)	18 (32.1)	12 (21.4)	29 (29.9)	32 (32.3)
≥ 3 to <5, mg/dL	97 (48.7)	53 (52.5)	29 (51.8)	34 (60.7)	56 (57.7)	52 (52.5)
≥5 mg/dL	23 (11.6)	8 (7.9)	9 (16.1)	10 (17.9)	12 (12.4)	15 (15.2)
MELD score, n	177	88	54	54	84	86
Mean (SD)	32.7 (6.63)	33.1 (6.16)	33.4 (6.02)	33.4 (6.34)	33.5 (6.23)	32.6 (5.47)
Child-Pugh-Turcotte class						

Class A (5–6)	3 (1.5)	2 (2.0)	0	1 (1.8)	5 (1.7)	0)
Class B (7–9)	68 (34.2)	32 (31.7)	9 (16.1)	10 (17.9)	100 (33.3)	29 (29.3)
Class C (10–15)	123 (61.8)	61 (60.4)	43 (76.8)	44 (78.6)	184 (61.3)	63 (63.6)
Missing	5 (2.5)	6 (5.9)	0	0	11 (3.7)	0
Baseline MAP (mm Hg), n	199	101	56	56	97	98
Mean (SD)	78.7 (12.08)	77.5 (9.36)	75.5 (11.42)	77.2 (13.58)	75.7 (11.82)	75.4 (10.54)
Bilirubin, n	189	97	56	55	94	95
Mean (SD), mg/dL	13.1 (13.52)	15.0 (15.58)	15.0 (13.6)	15.8 (15.1)	11.2 (10.67)	12.1 (12.79)
SIRS subgroup	84 (42.2)	48 (47.5)	—	—	28 (28.9)	30 (30.3)
Baseline MELD Score, n	177	88	54	54	84	86
Mean (SD)	32.7 (6.63)	33.1 (6.16)	33.4 (6.02)	33.4 (6.34)	33.5 (6.23)	32.6 (5.47)
Etiology of cirrhosis						
Alcohol	134 (67.3)	67 (66.3)	29 (51.8)	29 (51.8)	49 (50.5)	54 (54.5)
Hepatitis B	4 (2.0)	1 (1.0)	4 (7.1)	1 (1.8)	3 (3.1)	3 (3.0)
Hepatitis C	31 (15.6)	7 (6.9)	22 (39.3)	19 (33.9)	37 (38.1)	42 (42.4)
Primary biliary cirrhosis	5 (2.5)	3 (3.0)	2 (3.6)	1 (1.8)	4 (4.1)	3 (3.0)
Other	9 (4.5)	5 (5.0)	32 (57.1)	30 (53.6)	7 (7.2)	9 (9.1)
Cryptogenic (reverse)	6 (3.0)	3 (3.0)	—	—	8 (8.2)	5 (5.1)
Autoimmune hepatitis	10 (5.0)	5 (5.0)	2 (3.6)	3 (5.4)	1 (1.0)	1 (1.0)
Non-alcoholic steatohepatitis	42 (21.1)	24 (23.8)	2 (3.6)	5 (8.9)	8 (8.2)	7 (7.1)
Hepatocellular carcinoma, present	13 (6.5)	5 (5.0)	4 (7.1)	6 (10.7)	7 (7.2)	12 (12.1)
Esophageal varices, present	—	—	27 (48.2)	27 (48.2)	60 (61.9)	59 (59.6)
If yes, prior history?	30 (15.1)	21 (20.8)	8 (14.3)	13 (23.2)	18 (18.6)	27 (27.3)
Ascites, present	198 (99.5)	100 (99.0)	54 (96.4)	54 (96.4)	93 (95.9)	92 (92.9)

Data are presented as n (%), unless otherwise noted.

ITT, intent-to-treat; MAP, mean arterial pressure; MELD, Model for End-Stage Liver Disease; sCr, serum creatinine; SD, standard deviation; SIRS, systemic inflammatory response syndrome.
